# Supplementary material for: Pharmacokinetic analysis of intermittent rapamycin administration in early-stage Alzheimer's Disease
Source: GeroScience. 2025 Oct 5;48(3):3295–304. doi: 10.1007/s11357-025-01911-3 (PMC13355994; doi:10.1007/s11357-025-01911-3)
Supplement: Supplementary file 1 — Supplementary file1 (PDF 137 KB) [file 11357_2025_1911_MOESM1_ESM.pdf]

Supplementary Information

Pharmacokinetic analysis of intermittent rapamycin administration in early-stage Alzheimer's Disease

Helen Annervik Wallgren[1,2], Miia Kivipelto[1,4,5,6], Pontus Plavén-Sigray[2,3*], Jonas E. Svensson[1,2*]

1. Theme Inflammation and Aging, Karolinska University Hospital, Stockholm, Sweden

2. Department of Clinical Neuroscience, Karolinska Institutet and Stockholm Health Care Services, Region Stockholm, Stockholm, Sweden.

3. Neurobiology Research Unit, Copenhagen University Hospital, Copenhagen, Denmark.

4. Division of Clinical Geriatrics, Department of Neurobiology, Care Sciences, and Society, Karolinska Institutet, Stockholm, Sweden.

5. Ageing Epidemiology Research Unit (AGE), School of Public Health, Faculty of Medicine, Imperial College London, UK.

6. Institute of Public Health and Clinical Nutrition, University of Eastern Finland, Kuopio, Finland.

**Contributed equally to this work*

Corresponding author:

Jonas Svensson, Email: jonas.svensson@ki.se

Karolinska Institutet, Bioclinicum J4:14, Solnavägen 30, 171 64 Solna

Preparation of Standards and Sample

A nine-point calibration curve was prepared in blank human cerebrospinal fluid (CSF), with rapamycin concentrations ranging from 0.5 nM to 50 nM (approximately 0.5–50 ng/mL). Aliquots of 20 µL from each calibration standard were diluted with 80 µL of ice-cold methanol containing 50 nM warfarin as an internal standard. The samples were processed in a 96-well plate and centrifuged at 2465 × *g* at 4 °C for 20 minutes. A 5 µL aliquot from each well was injected into the LC-MS/MS system for analysis.
Following initial analysis, samples were evaporated to dryness and reconstituted in 20 µL methanol/water (1:1, v/v). A 10 µL aliquot was subsequently injected into the LC-MS/MS system.

Chromatographic Method

Chromatographic separation was performed on a BEH C_8 column (2.1 × 50 mm, 1.7 µm) at a controlled temperature of 60 °C. The mobile phases consisted of solvent A (10 mM ammonium formate in water) and solvent B (10 mM ammonium formate in methanol), both adjusted to pH 3.3.

Gradient elution was applied as follows: an initial composition of 10% B was maintained from 0.00 to 0.50 min, followed by a linear increase to 100% B at 1.20 min. This composition was held constant until 1.90 min, after which the gradient returned to 10% B at 2.00 min. The flow rate was set at 0.5 mL/min. A 10 mM ammonium buffer was necessary to effectively displace Na^+ and K^+ ion adducts, allowing exclusive formation of ammonium adducts, which are susceptible to fragmentation in the collision cell, enabling multiple reaction monitoring (MRM) detection.

Mass Spectrometry Conditions

Mass spectrometric detection was performed using a Xevo™ TQ-S micro triple quadrupole mass spectrometer (Waters Corp.) operating in positive electrospray ionization (ESI^+) mode. The instrument was set to MRM mode, with the following optimized parameters: capillary voltage of 3 kV, desolvation gas temperature of 100 °C, and a desolvation gas flow rate of 1000 L/h.

For quantitative analysis, MRM transitions were optimized for warfarin and rapamycin, including parent and fragment ions, cone voltages (Cone), and collision energies (CE). The warfarin transition was monitored at m/z 309.1596 → 163.0050, with a cone voltage of 28 V and a collision energy of 16 V. The MRM transitions for rapamycin were systematically optimized as follows: 931.37 → 351.13 (Cone: 5 V, CE: 25 V, quantifier ion), 931.37 → 452.09 (Cone: 10 V, CE: 30 V), 931.37 → 474.06 (Cone: 5 V, CE: 25 V), 931.37 → 492.08 (Cone: 10 V, CE: 20 V), 931.37 → 510.03 (Cone: 10 V, CE: 25 V), 931.37 → 524.06 (Cone: 10 V, CE: 30 V), 931.37 → 542.07 (Cone: 5 V, CE: 25 V).

Conclusions

Rapamycin is a cyclic compound with a molecular weight of 914.2 Da. During electrospray ionization (ESI), it predominantly forms Na^+ and K^+ adducts, which are resistant to collision-induced fragmentation (CID), making MRM-based detection challenging. This limitation was addressed by introducing ammonium-based buffers into the mobile phases to effectively displace metal ions. Additionally, the desolvation temperature was maintained at 100 °C to retain ammonium ions in solution. As a result, a stable rapamycin-ammonium ion complex (m/z 931.37) was generated, facilitating the development of a robust MRM method.

Using a standardized protein precipitation method with an organic solvent and a 2-minute LC-MS/MS method, an LLOQ of 20 nM (≈ 20 ng/mL) was achieved.

Given that the general sensitivity of the TQ-S micro system ranges from 0.5–10 nM, depending on the analyte and biological matrix, achieving lower LLOQ values may prove challenging with the current instrumentation.
